# Spatial Cues Provided by Sound Improve Postural Stabilization: Evidence of a Spatial Auditory Map?

**DOI:** 10.3389/fnins.2017.00357

**Published:** 2017-06-26

**Authors:** Lennie Gandemer, Gaetan Parseihian, Richard Kronland-Martinet, Christophe Bourdin

**Affiliations:** ^1^Aix Marseille Univ, CNRS, Perception, Representations, Image, Sound, Music (PRISM)Marseille, France; ^2^Aix Marseille Univ, CNRS, ISMMarseille, France

**Keywords:** auditory perception, postural stability, spatial sound, auditory landmark, auditory map

## Abstract

It has long been suggested that sound plays a role in the postural control process. Few studies however have explored sound and posture interactions. The present paper focuses on the specific impact of audition on posture, seeking to determine the attributes of sound that may be useful for postural purposes. We investigated the postural sway of young, healthy blindfolded subjects in two experiments involving different static auditory environments. In the first experiment, we compared effect on sway in a simple environment built from three static sound sources in two different rooms: a normal vs. an anechoic room. In the second experiment, the same auditory environment was enriched in various ways, including the ambisonics synthesis of a immersive environment, and subjects stood on two different surfaces: a foam vs. a normal surface. The results of both experiments suggest that the spatial cues provided by sound can be used to improve postural stability. The richer the auditory environment, the better this stabilization. We interpret these results by invoking the “spatial hearing map” theory: listeners build their own mental representation of their surrounding environment, which provides them with spatial landmarks that help them to better stabilize.

## 1. Introduction

Human postural control is a complex process involving multisensory integration. The classic sensory systems that are known to contribute to balance control are the visual, somatosensory and vestibular systems (Maurer et al., 2006). The auditory system is also thought to contribute to the process, but its contribution has been understudied, in spite of its great potential to provide spatial information. The aim of this paper is to further explore how the auditory system is involved into the postural control process, and more specifically, to better understand the role of the sound spatial cues in this process.

In an early study by Era and Heikkinen addressing the role of audition in postural control (Era and Heikkinen, 1985), the postural sway of young adults who had suffered hearing loss through exposure to noise at work was shown to be more pronounced than that of their unexposed peers. Several subsequent studies on various populations confirmed that lack of hearing (partial or total) was detrimental to postural control [e.g., in workers (Kilburn et al., 1992), in the elderly (Rumalla et al., 2015) and in congenitally deaf children (Suarez et al., 2007) or adults (Mangiore, 2012)].

These various assessments provide support for the hypothesis that auditory input plays a role in humans' postural regulation. However, few studies have addressed the question of how auditory information is used. Thus, the present paper focuses on the question of the specific role of audition in posture, and aims at identifying attributes of sound that could be useful for postural purposes.

In the sparse literature on sound and posture, most studies tended to show that auditory feedback can be used by human subjects to decrease postural sway. Several studies used static sound stimulations. Easton et al. (1998) had subjects stand in a tandem Romberg stance (heel-to-toe position) with two 500 Hz pure tone sound sources on both sides of their head, eyes open vs. eyes closed. The authors reported a 10% decrease in sway of in the presence of auditory cues. In a more recent study also involving subjects in tandem Romberg stance, subjects exposed to a pink noise sound source presented in front of them exhibited a 9% decrease in sway (Zhong and Yost, 2013) compared to no sound. Similarly, Vitkovic et al. (2016) found a reduction in sway for both normal-hearing and aided hearing-impaired subjects in the presence of a white noise sound source facing them.

A handful of other studies used sound in motion. Agaeva and Altman (2005), using sounds played by an arc of loudspeakers in the sagittal plane, found a small reduction in postural sway in the presence of a noise burst moving front/back. The study of Vitkovic et al. (2016) also showed that a pink noise sound source slowly moving from left to right on an 8-speakers semicircular array (passing behind the listener) helped subjects to decrease their postural sway. In another study conducted by Deviterne et al. (2005), sound stimuli were rotated around elderly subjects. Two types of rotating stimulations were compared: a “non-meaningful auditory message” (440 Hz continuous tone) and a “meaningful auditory message” (a short recorded story). In the “meaningful auditory message” condition, subjects were asked to carefully listen and were subsequently questioned about details in the story. The results showed that subjects were only stabilized in the meaningful condition. The authors concluded that the attention paid to the stimulus led subjects to take into account the spatial information it carried. A previous study by our team was conducted in a similar paradigm: subjects were asked to focus on a pink noise sound source rotating around them at various speed (Gandemer et al., 2014). Subjects also exhibited a significant decrease in sway in the presence of the rotating sound source (up to 30%) compared to absence of sound or a single static sound source facing them.

One emerging explanation for these effects is that sound sources provide acoustic landmarks through the spatial information they convey. Some researchers refer to an “auditory anchorage” effect (Deviterne et al., 2005), under the hypothesis that the spatial information provided by static sound sources can help subjects to construct a representation of the space surrounding them, and thus better stabilize. When we compare the results of our rotating sound study (Gandemer et al., 2014) (roughly 30% decrease in sway) to those of the static sound studies (roughly 10% decrease in sway), it is clear that moving sources (with the direction rather than the distance varying) lead to better stabilization. We assume that rotating sound provides more spatial information, as the acoustic cues vary and the source travels all around the subject. This raises the question of the relationship between the stabilization of subjects' postural sway and the amount of auditory information available. It seems plausible that the more spatial information conveyed by sounds, the greater the effect on postural control. Here, we explore this hypothesis by creating various auditory environments using static sound sources. The static sound studies presented above created very simple auditory environments, with rudimentary stimuli and/or apparatus: one loudspeakers delivering white noise (Zhong and Yost, 2013; Vitkovic et al., 2016), two loudspeakers delivering pure tones (Easton et al., 1998), etc.

In the present paper, we aim at using more complete means to control the auditory environment, in terms of the nature of sound stimuli, the control of sound reflections and the technology used to produce sound stimuli. We explore further how spatial auditory information is integrated into postural control, assuming that the postural sway of subjects depends on the quantity of spatial auditory information available and the nature of the sound sources. We chose to study the impact of ecological sound sources on subjects posture so as to ensure more natural listening conditions, in line with the ecological approach to auditory perception (Gaver, 1993). Then, we insisted on the control of sound reflections, by presenting the stimuli in various environments including an anechoic room, and on the way these sound sources were spatialized, using two different sound production technologies.

Two experiments were performed. The first compared the postural sway of subjects exposed to a simple auditory environment (created from 1, 2, or 3 static sound sources) in a normal room vs. in an anechoic room. Our goal was to determine to what extent subjects' degree of postural sway could be related to the quantity of auditory information available, and whether sound reflection could be informative for subjects involved in a postural task. In the second experiment, we enriched the auditory environment using two different techniques: either by adding sound sources or by synthesizing a 3D sound environment with sound field synthesis technologies. This latter technique exposed the subjects to a situation closer to natural listening.

## 2. Experiment 1: building an auditory environment

The goal of this first study was to determine whether the degree of postural sway could be related to the quantity of auditory information. We hypothesized that the richer the sound environment, the more subjects would be able to use auditory information to decrease their postural sway. Thus, we strictly controlled the spatial auditory information available: (1) the number of sound sources creating the auditory environment (1, 2, or 3 sources) and (2) the sound reflections in space.

### 2.1. Sound reflection

When propagating in space, sound reflects off obstacles, which creates a reverberated sound field composed of early reflections and diffuse fields. A substantial number of studies have addressed the effect of reverberation on spatial auditory perception. Their results suggest that sound reflections distort many acoustic cues, and could, for example, impede sound localization performance^1^ (Ribeiro et al., 2010). However, this negative effect has been found to be limited, especially if the reverberation is moderate (Shinn-Cunningham, 2003). Moreover, the information provided by reverberation is essential for distance estimation (Kolarik et al., 2015), and some studies also suggest that early reflections can provide information that enhances subjects' spatial perception (Ribeiro et al., 2010). Sound reflections are also useful when estimating the size and the shape of a room (Picinali et al., 2014). Stoffregen et al. (2009) even showed that blindfolded subjects were able to detect the movement of a room surrounding them solely from the reflected components of a sound field emitted inside the room, and to correlate their head movements with room motion.

Since sound reflections are known to impact the spatial perception of sound sources, the question arises of whether removing these reflections and reducing auditory information might affect subjects' postural sway. A handful of studies have compared subjects' postural sway in various auditory environments (Termoz, 2004; Kanegaonkar et al., 2012). Overall, postural sway was greater in an anechoic environment (free of reverberation) than in a classic reverberant space. Thus, in the present study we sought to compare postural sway in an anechoic vs. a reverberant space, hypothesizing that the anechoic environment would induce increased body sway.

### 2.2. Methods

#### 2.2.1. Subjects

The study group consisted of 35 young, healthy subjects: 22 men (age: 27.6 ± 4.7 years, min 22 max 37, height: 180.8 ± 7.2 cm) and 13 women (age: 25.8 ± 3.4 years, min 21 max 35, height: 166.5 ± 7.1 cm). None of the subjects reported either auditory or vestibular loss, or motor dysfunction. All of them participated on a voluntary basis; they signed an informed consent form prior to testing. This study was performed in accordance with the ethical standards of the Declaration of Helsinki (revised Edinburgh, 2000). The protocol was approved by the Ethics Committee of Aix-Marseille University.

#### 2.2.2. Stimuli and procedure

Subjects stood upright, barefoot, with their feet close together, on a force platform (Bertec, sampled at 250 Hz) measuring their postural sway. Subjects were blindfolded, to free their postural control system from the influence of visual input. They were instructed to maintain their position, without moving arms or legs, and to focus on sound stimulations, counting the number of sound sources surrounding them. Subjects were asked for their count between trials. This task was intended to ensure that the subjects focused on the sound sources and on their spatial locations.

**Figure 1:**
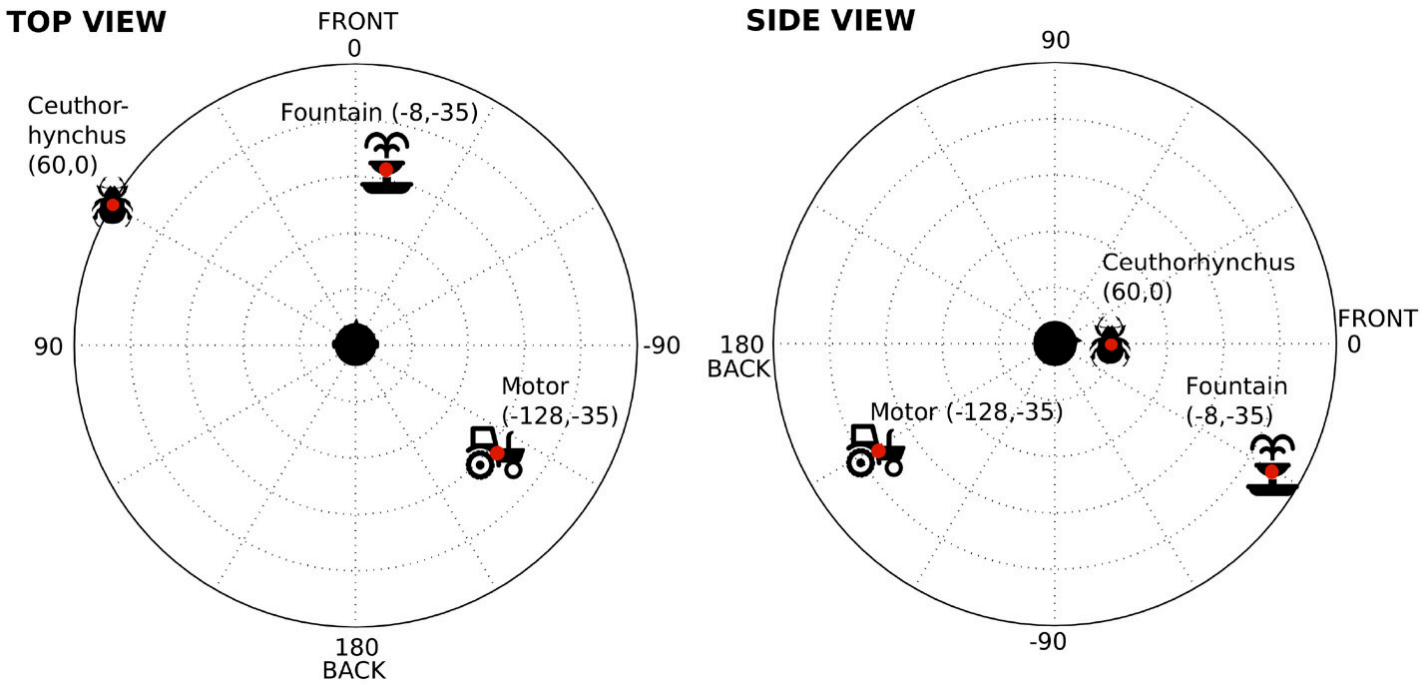
Nature and spatial positioning of the 3 sound sources used in Experiment 1. The dots show the precise position of the sources; spatial coordinates in parentheses (azimuth, elevation).

For the auditory stimulations, a simple auditory environment was built from three ecological sound sources, using samples played over loudspeakers. The precise nature and the position of the sound sources are shown in Figure 1. These three sound sources were chosen for their ease of localization (wide spectral content and/or grain), for their ease of discrimination (each new source sufficiently different from the others) and for their neutrality (no emotion conveyed by the sounds). They were positioned in such a way as to respect ecological criteria (plausible locations). For example, in real-life situations, sounds from a fountain or a car motor are usually located below our ears (negative elevation), whereas insect sounds generally come from the trees above or at the level of our heads (positive elevation). Human sound localization performances are known to be anisotropic, with listeners being for example more accurate when the source is presented in front of them, relative to the side (McCarthy and Olsen, 2017). But here, the sound sources were located at the same place during the whole experiment. The main point of this experiment was not to get the best accuracy in sound source localization, but rather to ensure that the subjects were able to perceive in space and discriminate the various sound sources surrounding them.

Subjects were exposed to 4 different auditory conditions:

- a reference condition without sound;
- 1 sound source (randomly chosen from the 3 available sources);
- 2 sound sources (randomly chosen from the 3 available sources);
- 3 sound sources.

Each of these auditory conditions lasted 32 s (including one second of fade-in and one second of fade-out) and was repeated 6 times. The order of presentation of the conditions was randomized. Thus, this experiment was realized with a counterbalanced design, in which every single participant was subjected to every single treatment, which were presented to each of them in a different order.

The experiment was duplicated in two different rooms: a normal room vs. an anechoic room. The normal room was a quiet studio, soundproofed with a 5 cm stone-wood layer covering the walls and a carpet on the floor. With these materials, the reverberation time was *RT*_60_ = 0.61 s at 250 Hz and *RT*_60_ = 0.41 s at 8,000 Hz, and the background noise was 24 dB_*A*_. This normal room was equipped with a 42-loudspeaker spherical array (described in Parseihian et al., 2015), of which only 3 loudspeakers were used in the experiment. The anechoic room was perfectly isolated from the outside (background noise: 17 dB_*A*_), and equipped with foam dihedrons on the walls so as to delete most of the sound reflection. These ensured nearly free-field conditions, where one sound source can be considered as a perfectly single sound source, “dry” of reverberation. Beside allowing us to control reverberation, comparing the two spaces also enabled us to verify whether the more perceptible background noise in the normal room affected subjects' body sway.

Each part of the experiment (normal vs. anechoic room) comprised 24 trials (4 auditory conditions x 6 repetitions) and lasted approximately 20 min. The subjects completed each part on separate days. Half of the subjects began with the normal room, and half began with the anechoic room part.

#### 2.2.3. Data analysis

The position of the Center of Pressure (COP) was calculated from the force platform force and moment data. Two descriptors were then calculated: area within the sway path and mean sway velocity. Area within the sway path is a global measure of the subjects' amplitude of sway. Amplitude of sway is related to the precision of postural control, whereas mean sway velocity stands for the efficiency of postural control (Perrin et al., 1999). Each parameter was averaged over the six repetitions of each condition and entered into a two-way repeated measures analysis of variance (ANOVA) with room and auditory condition as within-subject factors (2 and 4 levels respectively). Then, the Tukey's HSD test was used for all *post-hoc* analyses.

### 2.3. Results

Before presenting the postural results, it has to be noticed that subjects were precise and consistent in their counting. It means that they were able to perceive and discriminate the 3 sound sources in their surrounding space. We deliberately do not report the counting results here, as they are not the core of the present study.

#### 2.3.1. Area within the sway path

**Figure 2:**
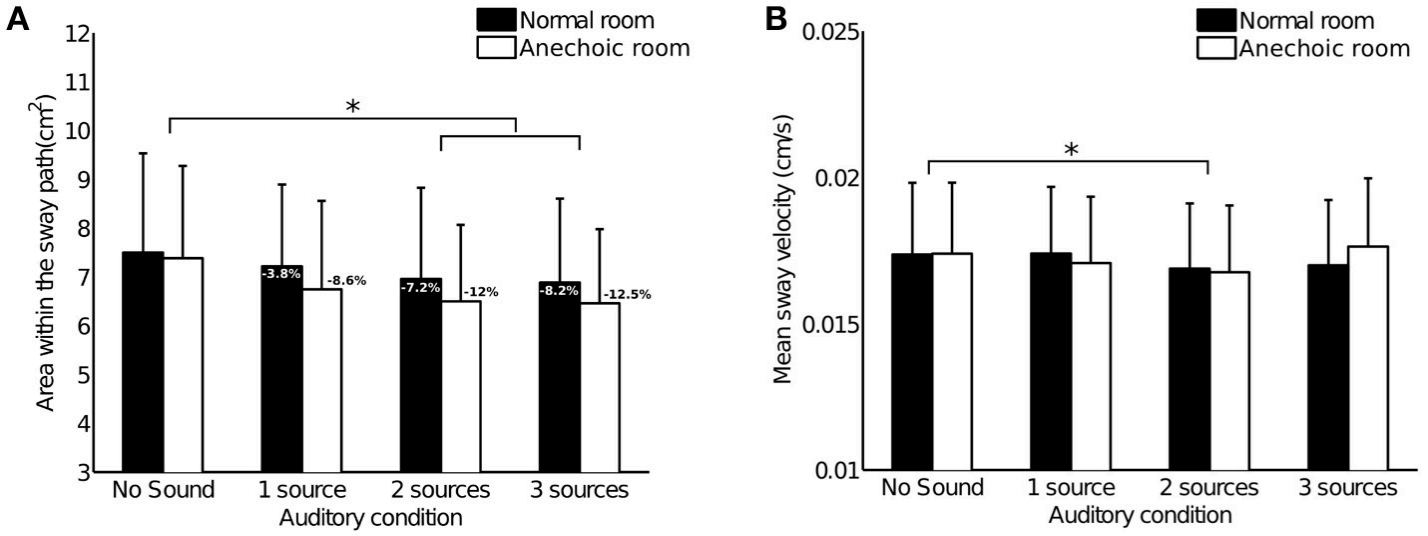
Results of Experiment 1. Bars represent the 95% confidence interval. Stars stand for a significant difference between the auditory conditions (*p* < 0.05). Analyses did not highlight significant differences between the two rooms nor significant interactions between the rooms and the auditory conditions. **(A)** Mean area within the sway path across subjects (*n* = 35). Percentages indicate decrease in sway comparing each condition with the “No Sound” reference condition. **(B)** Mean sway velocity across subjects (*n* = 35).

The results for area within the sway path presented in Figure 2A show a slight decrease in subjects' amplitude of sway as sound sources are added. This decrease in sway was found to be significant [$F_{\left(3,\;102\right)}=5.7804,p=0.0011,\eta^{2}=0.14531$]: Tukey's HSD *post-hoc* test highlighted a significant different between the “No Sound” condition and the “2 sources” and “3 sources” conditions (*p* < 0.01).

No significant differences between the two rooms [$F_{\left(1,\;34\right)}=1.4088,p=0.2435,\eta^{2}=0.03979$] or significant interactions between the rooms and the auditory conditions [$F_{\left(3,\;102\right)}=0.5181,p=0.6708,\eta^{2}=0.01501$] were found.

#### 2.3.2. Mean sway velocity

The results for mean sway velocity presented in Figure 2B do not show striking differences between conditions. The ANOVA nevertheless highlighted a significant difference between the auditory conditions [$F_{\left(3,\;102\right)}=2.8607,p=0.041,\eta^{2}=0.07761$], with mean sway velocity in the “2 sources” condition significantly lower than the velocity in the “No Sound” condition (*p* = 0.046).

No significant differences between the two rooms [$F_{\left(1,\;34\right)}=0.0230,p=0.8805,\eta^{2}=0.00068$] nor significant interactions between the rooms and the auditory conditions [$F_{\left(3,\;102\right)}=2.2641,p=0.08551,\eta^{2}=0.06243$] were found.

### 2.4. Discussion

Experiment 1 aimed to determine whether subjects' degree of postural sway could be related to the amount of auditory information available. Postural sway was compared in four auditory conditions with varying numbers of sound sources (0, 1, 2, or 3 sources). Moreover, the experiment was performed both in a normal room and in an anechoic room, to determine whether either the reflection of sound in space or background noise (both present in the normal room but not in the anechoic room) might also influence subjects' body sway.

#### 2.4.1. More sources, less body sway

Our results show that subjects' body sway significantly decreased in the presence of multiple static sound sources, when compared to a No Sound condition (mainly in terms of amplitude of sway, but also mean sway velocity, see Figure 2). The decrease in amplitude of sway reached 10% with three sound sources (average from both rooms). This magnitude of sway reduction is in line with results from other static sound studies [(Easton et al., 1998): 10% decrease in sway with two static sources, one placed adjacent to each ear; (Zhong and Yost, 2013): 9% decrease with one static source facing subjects]. Adding sources seemed to help subjects decrease their body sway, suggesting that the more auditory information provided, the better subjects can stabilize.

This observation confirms that spatial information provided by sound can be used to decrease body sway. The richer this information, the greater the decrease in body sway appears to be; however, to confirm this relationship, the auditory environment needs to be further enriched. Actually, the auditory environment created in this first experiment was relatively sparse, with a maximum of 3 sources. Moreover, this environment was not realistic: sound reflections were not implemented, and the directivity of the loudspeakers did not reflect real sources directivity. It can be hypothesized that adding more sound sources, or creating a more realistic and immersive environment, could lead to a greater decrease in subjects' body sway. We sought to test this hypothesis in the next experiment.

#### 2.4.2. No influence from the reverberated field

Surprisingly, the subjects did not exhibit postural differences between the normal room and the anechoic room conditions. We were expecting subjects to exhibit a greater sway in the anechoic room, as the auditory information available was impoverished (no sound reflections) compared to a normal room. This contradiction with our hypothesis may be explained by distinguishing the No Sound reference condition from the 3 conditions with sound sources.

First, in the No Sound reference condition, there was perfect silence in the anechoic room whereas background noise remained present in the normal room. This background noise is the only difference between the two conditions. Background noise, by definition, does not provide spatial information, as it is diffuse (not coming from a precise location in space). Some studies in the sound and posture literature have suggested that postural adjustments depend on the nature of the sound stimuli, and that background noise does not reduce subjects' body sway. For example, a recent study conducted by Gago et al. (2015) showed a disturbing effect of background noise on postural regulation of standing subjects. The authors compared, among other conditions, the postural regulation of subjects wearing or not wearing ear defenders. The subjects, positioned in a quiet laboratory with a normal level of background noise, exhibited greater postural sway without ear defenders than with ear defenders. The authors concluded that the background noise was not informative, and thus may have created more distraction than a total lack of auditory information. A similar result was obtained by Vitkovic et al. (2016), who showed that subjects swayed more in a normal room (in the presence of background noise) than in a soundproofed room, wearing ear defenders (complete lack of sound). A study conducted by Raper and Soames (1991) used background conversation sound stimuli delivered alternatively by 4 loudspeakers surrounding the subject. Subjects exhibited greater postural sway in the presence of these sounds compared to the reference without sound. In our case, while the slight background noise in the normal room did not seem to disturb the subjects, it did not seem to provide information either.

Then, in the 3 conditions with static sound sources in the anechoic room, the sources were played without any reflections, whereas in the normal room there was a slight reverberation (*RT*_60_ between 0.61 s and 0.41 s depending on the frequency band). Even though sound reflections have been shown to influence sound source localization (Shinn-Cunningham, 2003), as well as distance estimation (Kolarik et al., 2015), they did not seem to influence the postural regulation of our standing subjects. Thus, while the supplementary auditory information provided by sound reflections can be useful in a pure auditory perceptual task, it is not useful in our postural task. It may be that this postural task does not require as much precision in the treatment of auditory input as a localization task, which could explain why the moderate reverberated field in the normal room did not induce postural effects.

#### 2.4.3. Multisensory approach

However, the sound and posture literature contains two studies showing that sighted adults exhibited a greater sway in an anechoic environment than in a reverberant space, under no-sound conditions (Termoz, 2004; Kanegaonkar et al., 2012). Kanegaonkar et al. (2012) compared the body sway of subjects in a normal room vs. in an anechoic room, eyes open vs. eyes closed. They showed that with eyes open, subjects exhibited greater postural sway in a anechoic room than in a normal room. However, they did not find any significant difference between the two rooms when subjects had their eyes closed. Findings by Termoz (2004) on subjects with their eyes open were similar. The results of these two studies do not, therefore, contradict our study, where the subjects had their eyes closed. To explain our results, it is essential to bear in mind that posture is multisensory. The various cues provided by the different sensory modalities are weighted by the central nervous system, depending on environmental conditions (Assländer and Peterka, 2014). According to the quantity of sensory information available and the difficulty of the postural task, subjects may use sensory cues differently. In critical cases, sensory needs are transferred to modalities that are considered more reliable.

When the listener is in an anechoic room, blindfolded and without sound stimulations, he/she is deprived of both visual and auditory information that could allow him/her to build a mental representation of his/her surrounding space. In contrast, his/her proprioceptive, vestibular and somesthesic (mainly through plantar touch) inputs are fully available (Maurer et al., 2006). It is therefore possible that a sensory reweighting occurs, tipping the scales against vision and audition, and toward the other modalities. The subject may then rely on his/her own body map rather than a spatial map of his/her surrounding environment. This could explain why changing the auditory environment does not influence subjects' body sway, in the present experiment as well as in Kanegaonkar et al. (2012) (in the closed-eyes condition). In contrast, the listener with his/her eyes open has spatial information about his environment and can use these visual cues, which can be supplemented and combined with auditory cues. In this situation, subjects may give more weight to the visual and auditory modalities, which could explain why (Termoz, 2004; Kanegaonkar et al., 2012) observe that the deletion of auditory information (in an anechoic room) causes an increase in subjects' body sway.

Thus, we can hypothesize that in the absence of auditory and visual cues, subjects give greater weight to vestibular, proprioceptive and somatosensory inputs. One way to test this “transfer of sensory needs” hypothesis is to put the subjects on foam, thereby reducing the somatosensory feedback from plantar touch (Patel et al., 2011). If the subjects are more disturbed by the impoverishment of their auditory environment when they are standing on foam, it will provide support for the hypothesis that they transfer their sensory information needs to the somatosensory modality. We sought to determine this in Experiment 2.

### 2.5. Conclusions from experiment 1

In this first experiment, we showed that adding sound sources to the acoustic environment of a listener enables him to reduce his body sway. This confirms that subjects use the spatial information provided by static sound sources. However, adding moderate sound reflections does not have a significant impact on subjects' postural behavior, suggesting that sound reflections do not provide additional information that can be used to improve postural regulation.

In the present study, subjects reached a decrease in sway of about 10%. This decrease in sway, while in line with results from other static sound studies, is slight compared to our previous rotating sound study, in which subjects reached a decrease in sway of about 30% (Gandemer et al., 2014). In the present experiment, even in the 3-source conditions, subjects still had less auditory information available than with rotating sound (no variation in acoustic cues, and only three discrete spatial positions for sound). Yet their amplitude of sway slightly decreased when we enriched the auditory environment (adding sound sources). To determine the extent to which listeners' body sway might continue to decrease if we added further sound sources and enriched the environment, we performed the second experiment.

## 3. Experiment 2: enriching the auditory environment

The goal of this second experiment was to enrich the auditory environment of Experiment 1, seeking to determine whether this produced a better level of stabilization. We used two different techniques: (1) adding more sound sources, or (2) recording and playback in ambisonics an immersive auditory environment. Since in the Experiment 1 no differences were observed between the normal and the anechoic room, Experiment 2 was only performed in a normal room; however, the auditory conditions involved subjects standing on a normal vs. on a foam surface, to investigate the influence of a decreased plantar touch feedback on the auditory information integration.

### 3.1. Methods

The methods used for Experiment 2 were generally similar to Experiment 1. The main difference was the use of richer, more complex auditory stimuli in Experiment 2.

#### 3.1.1. Subjects

The study group consisted of 30 young, healthy subjects: 15 men (age: 28.1 ± 4.5 years, min 24 max 40, height: 175.9 ± 7.2 cm) and 15 women (age: 28.2 ± 4.9 years, min 21 max 42, height: 163.3 ± 5.0 cm).

None of the subjects reported either auditory or vestibular loss, or motor dysfunction. All of them participated on a voluntary basis; they signed an informed consent form prior to testing. This study was performed in accordance with the ethical standards of the Declaration of Helsinki (revised Edinburgh, 2000). The protocol was approved by the Ethics Committee of Aix-Marseille University.

#### 3.1.2. Stimuli and procedure

Subjects were instructed to stay still while focusing on the sounds, counting the number of surrounding sound sources, and to verbally report this count between trials.

**Figure 3:**
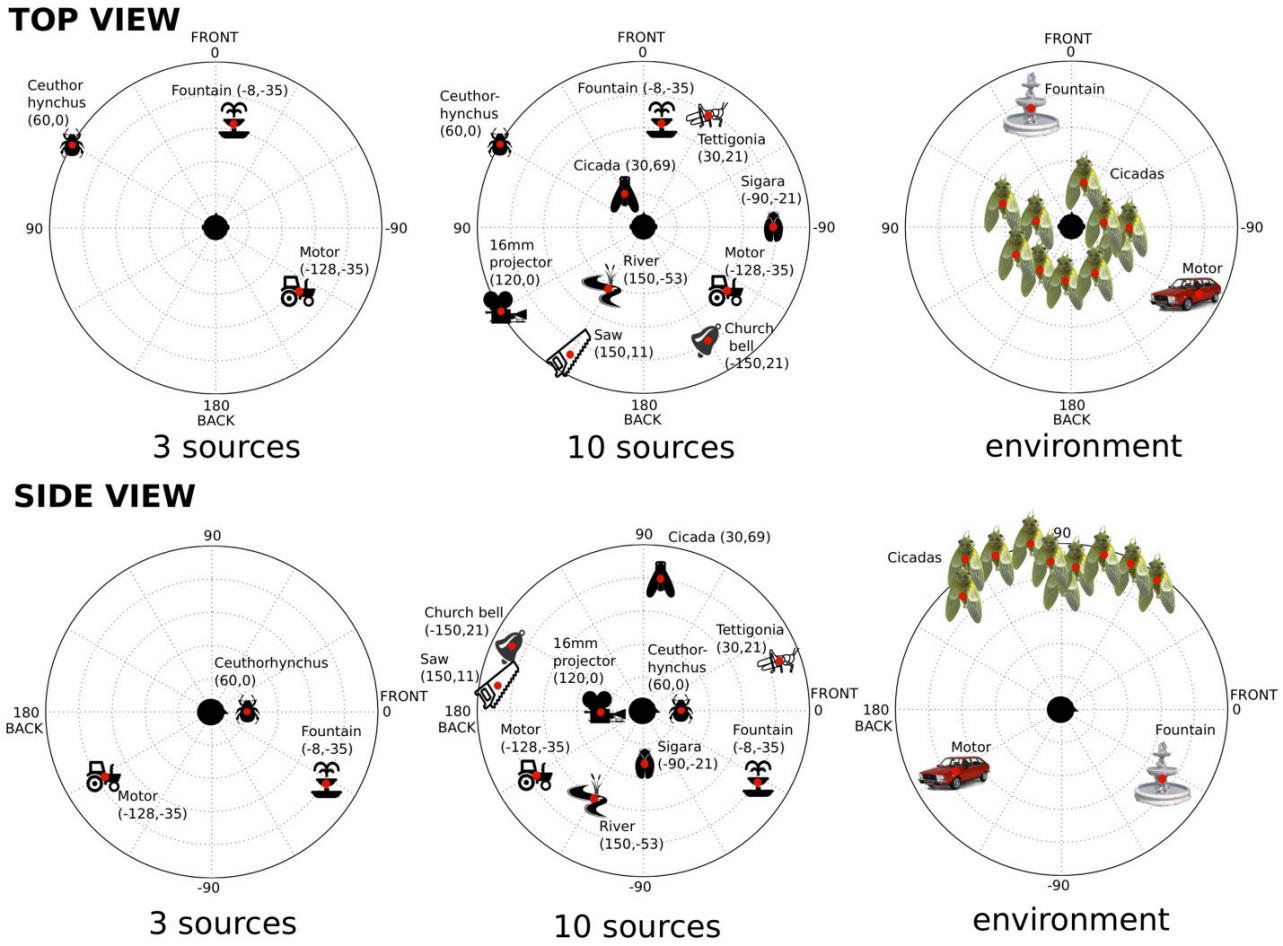
Nature and spatial positioning of the sound sources used in Experiment 2. The dots show the precise position of the sources; spatial coordinates in parentheses (azimuth, elevation).

When designing the auditory stimuli, it was decided to enrich the static auditory environment built in Experiment 1, using two different approaches. In the first, other isolated sources were added, using more samples played over other loudspeakers. Thus, in one auditory condition, we played 10 samples over 10 different loudspeakers all around the listener. The nature and the position of these sound sources are described in Figure 3. Just as in Experiment 1, the 10 sources were chosen for their ease of localization (wide spectral content and/or grain), for their ease of discrimination (each new source sufficiently different from the others) and for their neutrality (no emotion conveyed by the sounds). Source amplitude was set at a comfortable level allowing for the discrimination of the 10 different sources.

The second approach consisted in recording a real sound environment and then re-synthesizing it in our loudspeaker array using high order ambisonics spatialization techniques, as described in the next section.

Thus, we used four different auditory conditions:

- a reference condition without sound (background noise: 30 dB_*A*_);
- 3 isolated ecological sources (same condition as Experiment 1) (average amplitude: 45.5 dB_*A*_);
- 10 isolated ecological sources (average amplitude: 50 dB_*A*_);
- an immersive environment consisting of the same 3 ecological sources (fountain, car motor and cicadas) recorded and re-synthesized in ambisonics (this process is described in the next section) (average amplitude: 46.5 dB_*A*_).

The relative amplitude of each of these four auditory conditions was perceptually equalized in accordance with the perceived loudness, by ear by the experimenters. Each of these auditory conditions lasted 32 s (including one second of fade-in and one second of fade-out) and was repeated 5 times. The order of presentation of the conditions was randomized into a block of 4 conditions.

Subjects stood on two different surfaces, which were compared: normal (or firm) surface vs. foam surface. In the firm surface condition, subjects stood barefoot directly on the force platform, whereas in the foam surface condition, subjects stood on a piece of foam. One half of the subjects started the experiment standing on foam, and the second half standing on the normal surface. We were seeking to determine whether less somatosensory feedback (on the foam surface Patel et al., 2008) could favor the use of auditory input in the postural task, and thus could result in sound having more influence on posture.

The whole experiment comprised 40 trials (2 surfaces × 4 auditory conditions × 5 repetitions) and lasted approximately 45 min. In the middle of the experiment, the participant stepped off the platform and sat comfortably for at least 3 min.

The experiment took place in the normal room used in Experiment 1.

#### 3.1.3. Ambisonics synthesis of a realistic and immersive auditory environment

**Figure 4:**
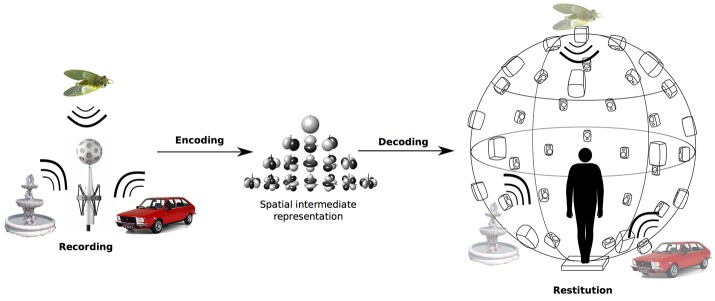
Ambisonics chain. The auditory scene is recorded on the spherical microphone array, encoded in ambisonics, and then decoded on the spherical loudspeaker array to reproduce the whole auditory scene.

This second experiment aimed to create a more realistic and immersive auditory environment using the ambisonics technique of sound recording and restitution. The complete ambisonics chain is represented in Figure 4; the immersive auditory environment was produced in several steps.

First, the auditory scene for our system was designed, an exterior scene using the same kind of sound sources as in Experiment 1: a fountain, cicadas singing in a tree, and a car motor. Then, a spherical microphone array (mhaudio Eigenmike em32, Meyer and Elko, 2002) was placed at the center of this auditory scene. The auditory scene was recorded, and the Eigenmike raw recording was then encoded in 4th order ambisonics. The encoding step consisted in decomposition of the sound field on a spherical harmonics basis, yielding an intermediate spatial representation of the sound field (Daniel, 2000). The next step was 4th order decoding on a loudspeaker array consisting of 42 loudspeakers equally distributed over a 3-meter diameter geodesic sphere surrounding the subjects. The system is described in Parseihian et al. (2015). The encoding and decoding steps were realized using Ircam SPAT software (www.forum.ircam.com) in a Max/MSP (www.cycling74.com) environment. Once the signal is decoded, the 42 loudspeakers re-synthesize the whole auditory scene at the center of the sphere. At the 4th order, for an area including an average head of the listener (radius *r*_0_ = 9 cm), the frequency cutoff of the ambisonics system is *f*_*c*_ = 2, 426 Hz (Oreinos and Buchholz, 2015).

We were thus able to recreate the full 3D sound environment in a more realistic and immersive manner than using isolated sources over separate loudspeakers. Even if the bandwidth of the soundfield produced in ambisonics is limited [beyond *f*_*c*_ = 2, 426 Hz, the soundfield is not perfectly recreated, which can induce perceptual distortions (Brungart, 1999)], the auditory environment recreated in ambisonics actually includes sound attributes not simulated in our other auditory conditions: directivity of the sound sources, sound reflection on the ground, etc.

#### 3.1.4. Data analysis

Subjects' sway was measured using the same Bertec force platform as in Experiment 1, but the sampling rate was 1,000 Hz. Here again, area within the sway path and mean sway velocity, calculated from the COP data, were averaged over the five repetitions of each condition and entered into a two-way repeated measures analysis of variance (ANOVA) with surface and auditory condition as within-subject factors (2 and 4 levels respectively).

### 3.2. Results

#### 3.2.1. Area within the sway path

**Figure 5:**
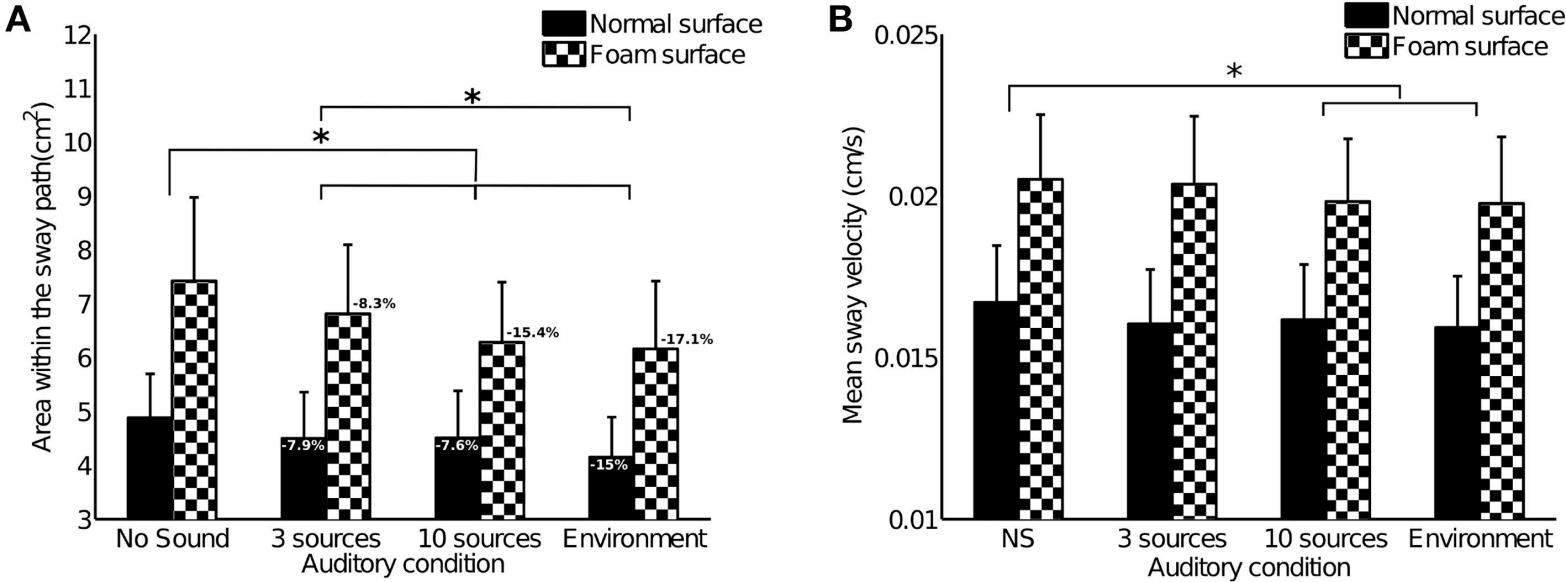
Results of Experiment 2. Bars represent the 95% confidence interval. Stars stand for a significant difference between auditory conditions (*p* < 0.05). Analyses highlighted significant differences between the two surfaces, but no interactions between surface and auditory condition. **(A)** Mean area within the sway path across subjects (*n* = 30). Percentages indicate the decrease in sway comparing each condition with the “No Sound” reference condition. **(B)** Mean sway velocity across subjects (*n* = 30).

The results for area within the sway path are presented in Figure 5A. Subjects exhibited far greater amplitude of sway when they stood on a foam surface. This result was found to be highly significant [$F_{\left(1,\;29\right)}=40.442,p<0.0001,\eta^{2}=0.5824$]. There were also significant differences between auditory conditions [$F_{\left(3,\;87\right)}=11.029,p<0.0001,\eta^{2}=0.2755$]. Tukey's HSD *post-hoc* test exhibited significant differences between the following conditions: “No Sound” vs. “3 sources” (*p* < 0.05), “No Sound” vs. “10 sources” and “Environment” (*p* < 0.001), “3 sources” vs. “Environment” (*p* < 0.05).

There were no significant interactions between the standing surface and the auditory conditions [$F_{\left(3,\;87\right)}=1.7045,p=0.1720,\eta^{2}=0.0555$].

#### 3.2.2. Mean sway velocity

The results for mean sway velocity are presented in Figure 5B. Subjects exhibited far higher sway velocity when they stood on a foam surface. This result was found to be highly significant [$F_{\left(1,\;29\right)}=33.229,p<0.0001,\eta^{2}=0.5340$]. There were also significant differences between auditory conditions [$F_{\left(3,\;87\right)}=3.8124,p=0.013,\eta^{2}=0.1162$]. Tukey's HSD *post-hoc* test exhibited significant differences between the following conditions: “No Sound” vs. “10 sources” and “Environment” (*p* < 0.05).

There were no significant interactions between the standing surface and the auditory conditions [$F_{\left(3,\;87\right)}=1.0286,p=0.3840,\eta^{2}=0.0343$].

### 3.3. Discussion

In this Experiment, the static auditory environment from Experiment 1 was enriched in two ways, to assess how subjects might be using the spatial information provided by sounds and, more importantly, to determine the sound parameters useful for the postural system. We hypothesized that the richer the auditory environment, the more subjects would be able to stabilize their body sway. We also compared the body sway of subjects standing on foam vs. on a firm surface, to determine whether reducing plantar tactile input might lead to sensory reweighting in favor of the auditory modality.

#### 3.3.1. Foam vs. firm surface

First, we compared subjects' postural sway when standing on a normal surface vs. a foam surface. The foam surface decreases plantar tactile feedback (Patel et al., 2011), an important sensory input in the upright stance (Kavounoudias et al., 1998). In postural control, it is known that modification of the availability of sensory inputs leads to sensory reweighting, with the more reliable sensory inputs given a greater weight (Assländer and Peterka, 2014). Thus, it has already been shown that when subjects are placed on foam, a sensory reweighting occurs in favor of vision (Patel et al., 2008). Our experiment sought to determine whether the foam surface condition would also lead to sensory reweighting in favor of audition, with subjects transferring part of their sensory needs to the auditory input.

When subjects stood on a foam surface, our results first show that their amplitude of sway and mean sway velocity were significantly greater than on a normal surface, as has been found in numerous studies (for example, Patel et al., 2008). In contrast, there were no significant interactions between surface of support and auditory condition, which means that subjects' response to the auditory conditions was the same on both surfaces. When standing on foam, the subjects still used the auditory information in the same way as on the normal surface, and did not seem to compensate for the lack of tactile feedback by an augmented use of the auditory environment.

However, we cannot conclude that there was no sensory reweighting. Indeed, there are numerous somatosensory input not related to plantar touch, which were not impaired by the foam surface and may have been used to compensate for the decrease of plantar touch feedback. The “standing on foam” conditions may not have been challenging enough to reach a sensory threshold, and a reweighting in favor of the auditory modality. A good way to further decrease the somatosensory information would be to use a platform whose angular position is coupled to subjects' hip angular position, in a “body-sway referenced" way (see, for example, Mergner et al., 2005).

#### 3.3.2. Richness of the auditory environment and stabilization

Next, our results show that subjects' amplitude of sway was smaller in all the conditions with sound, when compared to the “No Sound” reference condition. Moreover, subjects exhibited significantly better stabilization in the immersive “environment” condition (over 15% decrease in sway) than in the other auditory conditions (“3 sources”: roughly 8% decrease in sway; “10 sources”: 8 to 15% decrease in sway). In this “environment” condition, the sound stimuli consisted of a real 3D auditory environment, recorded and re-synthesized via ambisonics. This was the richest auditory condition, providing the greatest quantity of spatial information: in addition to direct sound, the ambisonics reproduction of the real auditory environment even included reflections from the sound sources. The ambisonics approach also faithfully reproduced the real sources' width and directivity. Source widths were therefore greater than for loudspeaker reproduction, with for example the cicadas occupying a large portion of the upper hemisphere. This contrasts with the “3 sources” and “10 sources” conditions, where each source's width and directivity were replaced by those of the loudspeaker reproducing it. Thus, more than the number of sound sources composing the environment, how those sounds represent the 3D space around the listener seems to be the key factor of the stabilization observed.

These results also show that subjects' stability improved in a homogeneous auditory environment compared to an environment consisting of isolated sources. We can draw a parallel with vision: watching the whole visual scene leads to better stabilization than staring at a precise point in the scene (Laurens et al., 2010).

Another way of enriching the auditory stimulation would be to let the subjects freely move their head. Indeed, there is a greater richness in auditory cues when the sound sources are moving, as mentioned in the introduction. When the listener is moving the head, he/she is producing moving auditory cues, with the additional advantage of having the congruent motor efferent information. It is conceivable that moving the head with auditory cues would demonstrate even greater postural benefits. Testing it would require more sophisticated movement tracking than the single force platform.

Thus, Experiment 2 confirms the intuition from Experiment 1: the richer the auditory environment (in terms of spatial information conveyed by the surrounding sounds), the better the stabilization of the listener. We now form our hypothesis about how this stabilization occurs.

#### 3.3.3. Cognitive map of the acoustic environment

In this experiment, we argue that the subjects built their own mental representation of the acoustic space from the sound stimuli [a “hearing spatial map" (Kanegaonkar et al., 2012; Vitkovic et al., 2016)], and used the static sound sources as auditory landmarks which helped them to decrease their postural sway. A mental representation of the surrounding space, also called a “spatial image", can be produced solely from sensory inputs (vision, audition, and/or touch) (Giudice et al., 2013). Most studies on spatial mental images built through sound were conducted on blind people. They show that the auditory information can suffice to build a precise and metrically accurate spatial map of the environment (Afonso et al., 2010).

To verify that listeners are building this spatial map of the acoustic space, we could set up a stable acoustic space and then suddenly disturb it (for example, tilting it). This kind of perturbation would conflict with the putative representation of space, inducing postural destabilization of the listener. We could expect to observe postural perturbations in the same direction as the auditory space perturbation, as has been observed with visual stimuli through a wide variety of paradigms (Peterka and Benolken, 1995): swinging room, tilting room, projected displays simulating a moving visual wall, etc.

## 4. Conclusion and perspectives

The results of these two studies provide supplemental evidences that spatial information conveyed by sound can be used by human subjects to help maintain postural stability. They also highlighted that the richer the auditory environment, the greater the decrease of subjects' amplitude of sway. It confirms our initial assumptions and suggests that auditory spatial perception plays a fundamental role in the postural process. To explain the influence of sound on posture, we hypothesize here that these auditory cues make the construction of a spatial mental map of the surrounding auditory space possible; then, the subjects can stabilize with respect to this spatial map. However, sound reflections (in an anechoic vs. a slightly reverberant space) are not shown here to impact postural behavior. This suggests that not all attributes of sound can be used in a postural task, even those that are clearly useful in a pure auditory task.

Using a multisensory approach, we also found that listeners exhibited the same behavior on a firm vs. on a foam surface, meaning that modifying their somatosensory input did not modify the way they were using sound.

The results obtained in both experiments confirm that sound can play a significant role in the improvement of postural control. This opens the way to numerous applications in various fields, such as sport, rehabilitation or sensory substitution. Auditory-biofeedback systems are a good example of such an application: these systems aim at the sonification of postural displacements (Chiari et al., 2005). They have been shown to be effective in improving subjects' postural control. The next step could be to create an interaction between movement and auditory stimuli. Future research might track subjects' motion and modify auditory stimuli in real time in accordance with subjects' movements. This could serve to disturb auditory landmarks, which could be moved in a manner congruent or incongruent with the listener's movements.

## Author contributions

LG, GP, RK, and CB contributed to the design of the experiment. LG managed the operation of the tests. LG, GP, RK, and CB contributed to the writing of the paper.

### Conflict of interest statement

The authors declare that the research was conducted in the absence of any commercial or financial relationships that could be construed as a potential conflict of interest.

## Footnotes

^1^This negative effect is natural and expected, given that strong reflections can have amplitudes similar to that of the direct signal, but different directions of arrival.
